# Potential of AI-based diagnostic grading system for knee osteoarthritis

**DOI:** 10.3389/fmed.2025.1707588

**Published:** 2025-10-30

**Authors:** Saman Shahid, Aamir Wali, Aatir Javaid, Shahid Zikria, Onur Osman, Jawad Rasheed

**Affiliations:** ^1^Department of Sciences and Humanities, National University of Computer and Emerging Sciences (NUCES), FAST Lahore Campus, Lahore, Pakistan; ^2^Department of Data Sciences, National University of Computer and Emerging Sciences (NUCES), FAST Lahore Campus, Lahore, Pakistan; ^3^Department of Orthopedic Surgery, University College of Medicine, The University of Lahore (UOL), Lahore, Pakistan; ^4^Department of Computer Science, Information Technology University (ITU), Lahore, Pakistan; ^5^Department of Electrical and Electronics Engineering, Istanbul Topkapi University, Istanbul, Türkiye; ^6^Department of Computer Engineering, Istanbul Sabahattin Zaim University, Istanbul, Türkiye; ^7^Department of Software Engineering, Istanbul Nisantasi University, Istanbul, Türkiye; ^8^Research Institute, Istanbul Medipol University, Istanbul, Türkiye; ^9^Applied Science Research Center, Applied Science Private University, Amman, Jordan

**Keywords:** computational imaging, healthcare AI, disease prediction, AI-based diagnosis, transfer learning, computer-aided diagnostic system, radiology

## Abstract

**Background:**

Knee osteoarthritis (KOA) is a progressive musculoskeletal disorder and a leading cause of disability worldwide. Early and accurate diagnosis is crucial for timely intervention; however, conventional manual grading using radiographs is prone to variability. Artificial intelligence (AI)-based computer-aided diagnostic (CAD) systems offer potential to improve detection and grading accuracy.

**Objective:**

This study aimed to develop and evaluate an AI-based diagnostic grading system for KOA using X-ray imaging and transfer learning techniques, with the goal of assisting clinicians and medical trainees in early and precise diagnosis.

**Methods:**

An experimental cross-sectional study was conducted using 301 radiographs (602 knee images) collected from the Social Security Teaching Hospital, Lahore. The dataset included Kellgren–Lawrence (KL) grades 0–4, with labeling based on pain observation and expert orthopedic assessment. Image preprocessing involved binary thresholding, morphological operations, knee isolation, normalization, and zero-padding. Transfer learning with DenseNet-121 served as the base network, augmented by convolutional and fully connected layers. Performance was evaluated against other deep learning architectures (DenseNet201, ResNet50, MobileNet) and classical machine learning algorithms (SVM, decision tree, random forest). Metrics included accuracy, area under the curve (AUC), precision, and recall.

**Results:**

DenseNet-121 demonstrated the most robust performance among the tested models, achieving an accuracy of 68.85%, an AUC of 85.67%, a precision of 68.33%, and a recall of 67.21% on the independent test set. Comparative models, including DenseNet201 and MobileNet, exhibited lower accuracies (≈60 to 61%) and AUCs (≈80 to 83%). Machine learning approaches underperformed, with a maximum accuracy of 55.73%. The primary challenges included dataset imbalance and the difficulty in distinguishing between grade 0 and grade 1 due to overlapping radiographic features.

**Conclusion:**

The proposed AI-based CAD system shows promise for supporting KOA diagnosis and grading in clinical practice, particularly for training junior clinicians and radiologists. Despite limitations of dataset imbalance and restricted single-center data, transfer learning with DenseNet-121 achieved reliable performance. Future work should focus on expanding datasets to encompass diverse populations, incorporating multimodal inputs, and validating generalizability across various clinical settings. This approach highlights the growing role of AI in musculoskeletal imaging and its potential to enhance early disease detection and patient care.

## 1 Introduction

Osteoarthritis (OA) is a common chronic joint disease with numerous risk factors (1, 2), often affecting weight-bearing joints (3, 4), particularly the knee (5). OA can impact individuals of all ages but is more common in older adults, particularly in women (6). Symptoms include knee pain, stiffness, and swelling, typically aggravated by activity and relieved by rest or inactivity (7). OA in the knee (KOA) can lead to joint deformity, discomfort, and functional impairment, contributing to disability worldwide (8, 9). KOA is prevalent in approximately 10% of adults over 65 (10), with factors like age, gender, hormonal changes, genetics, obesity, excessive physical activities, and injury contributing to its development. It is anticipated that by 2050, approximately 1.3 billion people worldwide will be affected by it (11–14). Early detection and treatment can help slow its progression and improve the quality of life for older people.

Diagnosing and treating knee KOA is complex due to multiple risk factors, and early detection remains challenging (15). Plain radiographs are commonly used to evaluate features such as joint space narrowing, osteophyte formation, and subchondral changes. The Kellgren–Lawrence (KL) grading system, introduced in 1957 (16) and later recognized by the WHO (17, 18), is the standard for classifying OA severity from grade 0 (healthy) to grade 4 (severe). However, studies measuring joint space width have shown inconsistent results. To address these limitations, computer-aided methods using machine learning (ML) offer a promising approach for automating KOA severity assessment (19).

Over the last decade, several techniques for knee joint detection and categorization by KL grade have been developed. Schiratti et al. (20) conducted binary classification on knee MRI images, achieving an Area Under the Curve (AUC) score of 72%. However, the study primarily focused on predicting the current state of the disease rather than forecasting future disease progression. This limitation restricts the generalizability of the findings to long-term outcomes. Moustakidis et al. (21) employed ML and Dense Neural Networks (DNNs) in their study. They trained the DNN model using data from 4,796 patients, achieving a binary accuracy rate of 79.6%, albeit lower than what’s typically expected in binary datasets. On the other hand, Mahum et al. (22) employed hybrid features of CNN, along with Local Binary Pattern (LBP) and CNN with Histogram of Oriented Gradient (HOG), for feature extraction, and utilized a support vector machine (SVM) for the classification task. Convolutional Neural Networks (CNNs) have shown immense promise in computer-aided diagnosis (CAD), sometimes achieving human-level performance (23–26). Similarly, Leung et al. (27) used transfer learning techniques for the classification. However, none of them separate the knee from single X-rays. They all used preprocessed datasets, and the model performed very well according to them.

In this study, we present an end-to-end AI-based computer-aided diagnostic (CAD) system for the automatic detection and severity grading of knee osteoarthritis (KOA) from raw bilateral X-ray images. KOA is a prevalent musculoskeletal condition that significantly impacts knee function (28) and quality of life, yet its manual diagnosis can be challenging, particularly for junior clinicians. To support clinical evaluation, we developed a system with several key contributions. First, we introduce a newly collected dataset comprising 602 knee images from 301 patients across five KL-grade classes—the first of its kind from our clinical setting. Second, we designed an automated pipeline for knee isolation from bilateral radiographs, addressing a preprocessing step often neglected in previous studies. Third, we implemented and compared multiple machine learning and deep learning architectures, identifying a fine-tuned DenseNet-121 model as the most effective. This model, trained with data augmentation to enhance robustness, demonstrates the feasibility of utilizing AI to support radiologists and trainees in achieving more consistent KOA severity assessments.

Accurate disease prediction is of paramount importance in the field of medicine, particularly when dealing with complex and challenging datasets. We applied several approaches to a complex labeled dataset, with the ultimate goal of improving KOA prediction. This work can provide clinicians with more precise disease staging, enabling earlier intervention for risk factors like disability and functional impairment. Patients’ quality of life can be significantly enhanced as a result. Caregivers often seek medical advice only after the disease has progressed to late stages, highlighting the need for early and precise diagnosis. This research addressed this challenge by developing an automated computer-aided diagnostic system that leverages AI tools and decision models. The major goal was to accurately diagnose KOA at an early stage, potentially alleviating the burden of this prevalent disorder and its related problems. The dataset, obtained with permission from the Department of Orthopedic Surgery and Traumatology at the Social Security Teaching Hospital, Lahore, includes patients with KOA grades ranging from 0 to 4, where 0 represents normal cases without osteoarthritis, while grade 4 represents cases with severe osteoarthritis. The study employs Transfer Learning (TL) techniques to extract AI-based markers, enabling the automated prediction of the disease’s severity.

## 2 Materials and methods

### 2.1 Study design and setting

A retrospective cross-sectional study was conducted at the Department of Orthopedic Surgery and Traumatology, Social Security Teaching Hospital, Lahore, and the National University of Computer and Emerging Sciences (NUCES), Lahore, Pakistan. The data was collected from the records of 1st October 2023–31st March 2024. The research conforms to the requirements recommended by the STROBE initiative for cross-sectional studies. Experiments related to AI models were conducted. Prior ethical approval was taken from the Hospital. The retrospective clinical and X-ray data were collected with informed consent from the patient. While collecting the dataset, we adhered to the established ethical standards. A total of 301 patient X-rays were utilized in this study, resulting in the extraction of 602 knee images. Among these patients, 18 had grade-0 (no disease), and 67 had grade-1 (doubtful joint space narrowing, possible osteophytic lipping) based on radiographic assessment by an orthopedic surgeon, using the original Kellgren–Lawrence criteria (16). 105 had grade-2, 126 had grade-3, and 86 had grade-4 knee conditions. Our dataset was selected from the Social Security Teaching Hospital as it represents a typical clinical population encompassing the full spectrum of KOA severity from early to advanced stages. To prevent data leakage, we ensured that all images from a single patient were contained within either the training or test set, never split between them.

### 2.2 Inclusion and exclusion criteria

To ensure the relevance and integrity of the research findings, specific inclusion and exclusion criteria were applied in the selection of participants for this study. The inclusion criteria encompassed individuals aged between 40 and 80 years who reported experiencing pain in one or both knees and had a documented history of persistent knee pain for a minimum duration of 3 months. Patients who had recently experienced trauma, had surgical operations around the knee joint, or were diagnosed with knee septic arthritis or knee rheumatoid arthritis were excluded from the research.

### 2.3 Dataset

The dataset comprised 2D X-ray images, classified into five distinct categories. A senior orthopedic surgeon labeled the data for the precise classification of the ground truth. With a total of 301 samples available in PNG format, each image included both knee joints. The size of the dataset was found to be sufficient for the experimentation. The gender distribution among patients is 162 females and 139 males. Considering population data, the collective average age stands at 59.85 ± 11.4 years. Patient age and gender details are indicated by the data operator in the upper left corner of the X-ray images. The dataset comprised five classes, where class 0 indicated the absence of disease, while classes 1 through 4 represented the stages of the disease, progressing from low to high, respectively.

Each sample included two separate knees, labeled separately; therefore, an additional preprocessing step was used to ensure accurate label prediction for each knee. Our approach involved algorithmically separating the knees within the images before model input. Given the varying sizes of the dataset images, our preprocessing pipeline encompassed resizing, cropping, and padding operations. These steps were integrated into the preprocessing methodology outlined in the section below of this study.

### 2.4 Proposed work

**Figure 1:**
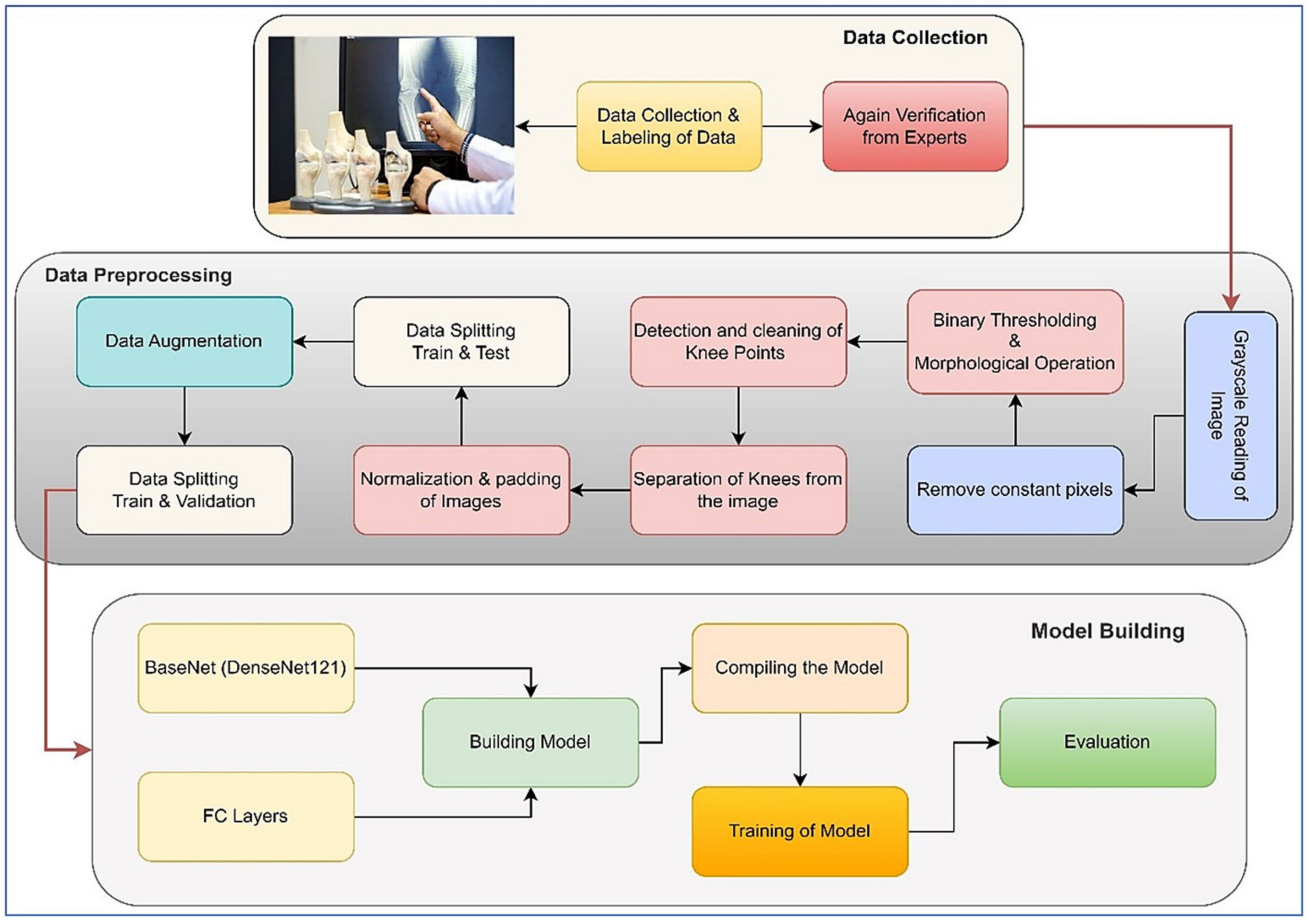
Methodology flow diagram of proposed work.

In the outlined methodology, the X-ray images inherently featured two knees. To isolate each knee, image-processing steps were implemented to handle the pipeline. Initially, pixel intensities were enhanced through sharpening. After that, a binary thresholding technique was employed to eliminate background pixels. Following that, a morphological opening operation was used to successfully separate the prominently highlighted area, which largely corresponded to the knee region due to the predominance of bone structures in the X-rays. Another thresholding step was then performed to determine the start and end columns of each knee. Padding was done after the images were normalized. These processed images were then utilized for model training purposes. Two significant challenges were addressed in this study. The first challenge entailed dealing with an unbalanced dataset, while the second involved constructing an equal number of image slices for each patient. The visual representation of our approach is shown in Figure 1, which illustrates each step of the methodology. Further elaboration on each step can be found in the subsequent subsections. The following sections provide comprehensive insights into the pre-processing procedures and the specific details concerning the network’s architecture.

### 2.5 Data samples

**Figure 2:**
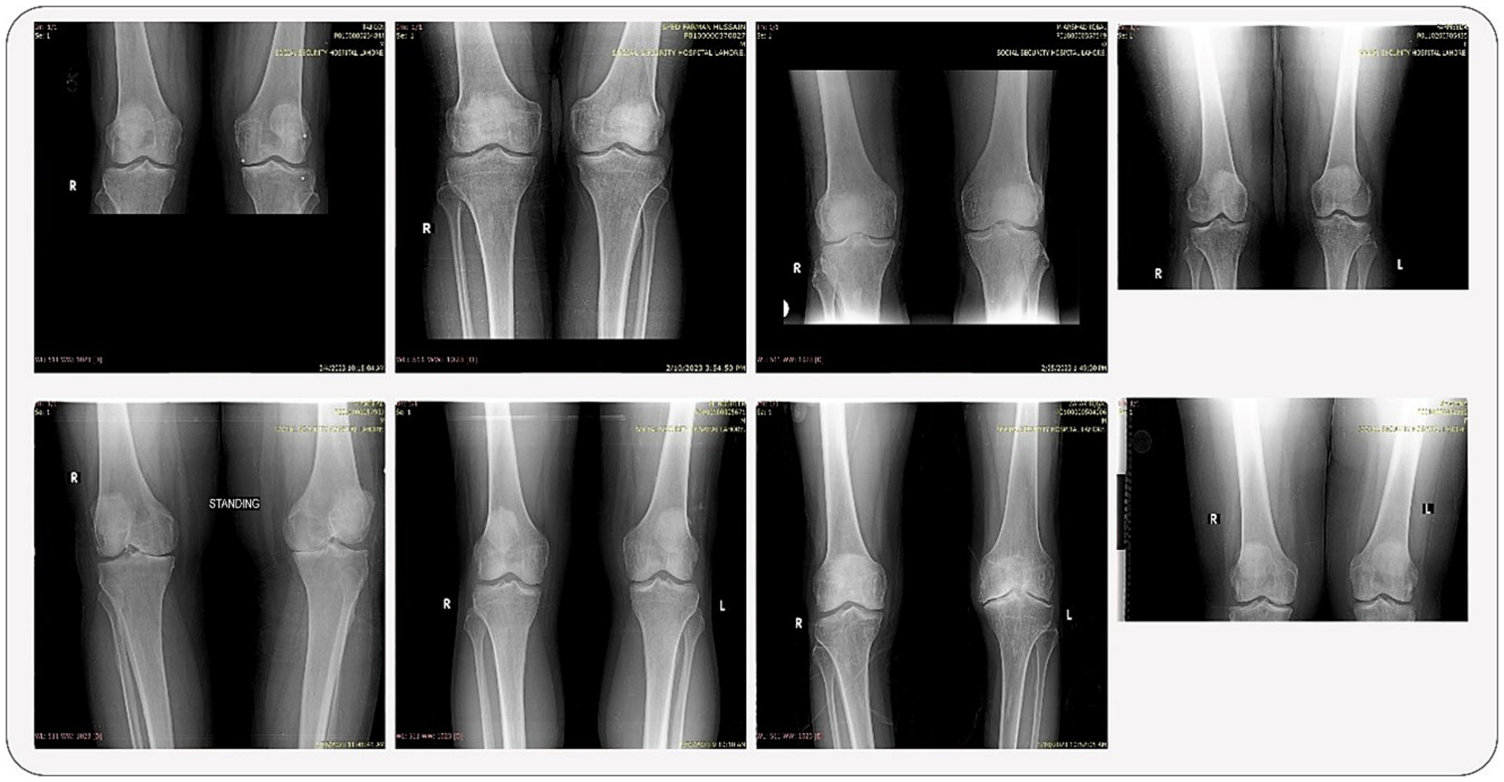
Some random samples from the original dataset.

This section provides a brief overview of the dataset’s composition and variety. Some samples from the dataset are displayed, offering a visual depiction of X-rays with varying grades. With images that show different levels of illness progression, these samples demonstrate the dataset’s heterogeneity. These samples give readers a first sense of the intricacy of the dataset, laying the groundwork for the subsequent approaches and conclusions described in the study. The samples are shown in Figure 2.

### 2.6 Image preprocessing and knee isolation

The primary challenge in utilizing the bilateral knee X-rays was the need to isolate individual knees for classification. We developed an automated image-processing pipeline for this purpose, with the overall workflow illustrated in Figure 1.

**Figure 3:**
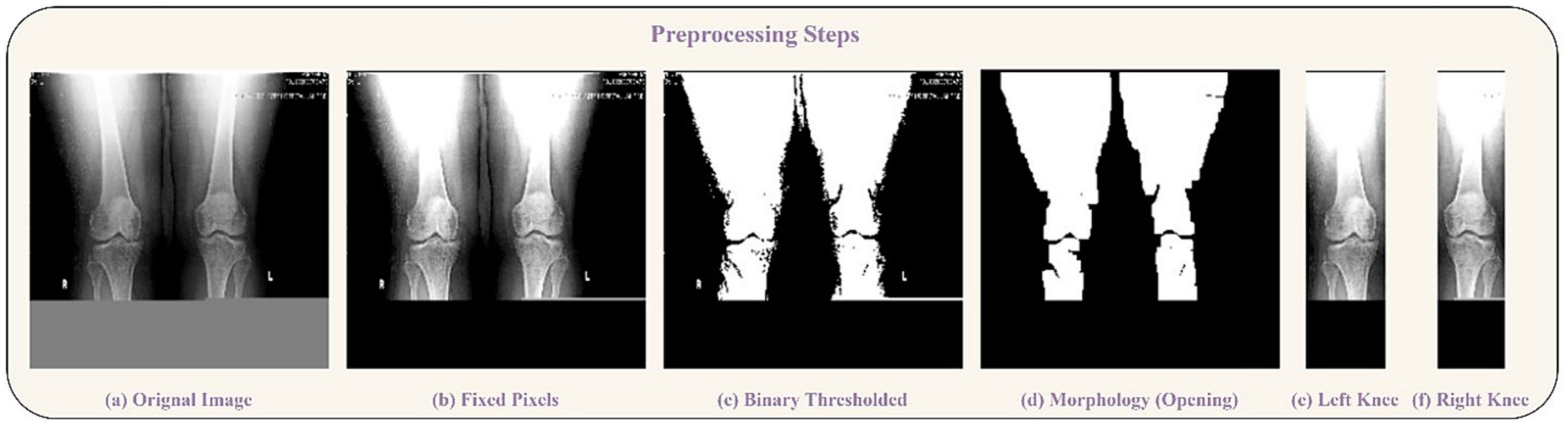
Preprocessing steps visualization. **(a)** Original Image, **(b)** Fixed Pixels, **(c)** Binary Thresholded, **(d)** Morphology (Opening). Segmentation of knees: **(e)** Left Knee, **(f)** Right Knee..

**Figure 4:**
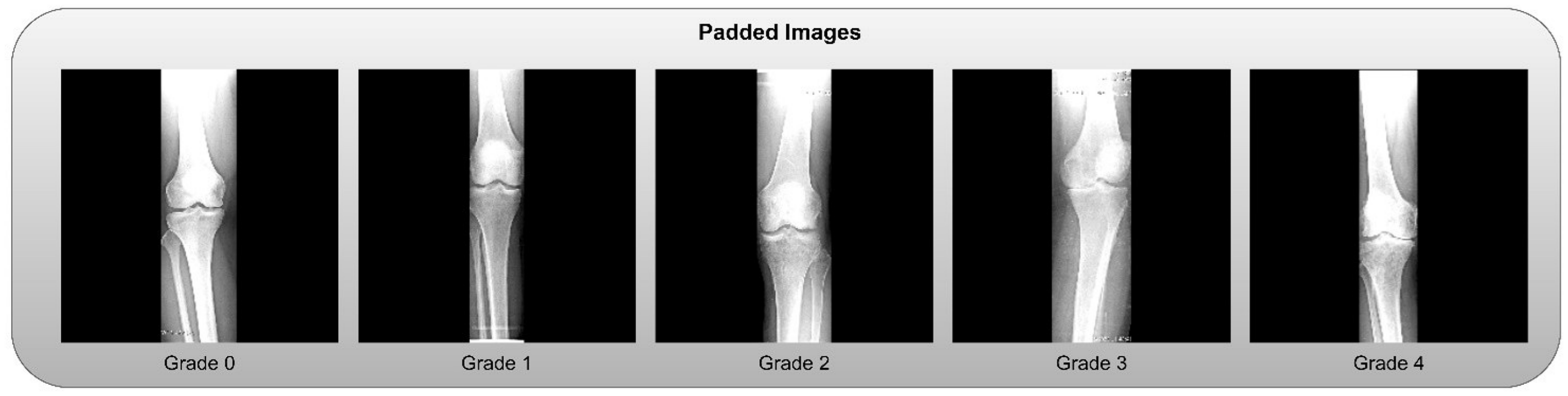
Clipped knees with padded slices.

1. Resizing and Color Conversion: All original images (see samples in Figure 2) were first resized to a uniform dimension of 224 × 224 pixels and converted to grayscale.
2. Knee Segmentation via Thresholding and Morphology: We employed binary thresholding using the OTSU algorithm to create a mask separating the radiopaque bone structures (foreground) from the background. A morphological opening operation was then applied to this mask to remove small noise artifacts and disconnected pixels. In cases where thresholding yielded suboptimal segmentation, a pixel correction strategy was used, replacing rows of identical pixel values with zeros. The results of these steps are visualized in Figure 3.
3. Knee Detection and Cropping: The processed morphological image was analyzed column-wise to detect the start and end points of each knee joint by identifying columns with non-zero pixel values. This process was repeated to capture both knees, which were then cropped into individual images.
4. Post-processing for Model Input: The cropped knee images, which were of variable sizes, underwent two final steps:

- Zero-Padding: To standardize dimensions without distorting the aspect ratio, zero-padding was applied to the sides of the cropped slices. Examples of the final padded knee slices are shown in Figure 4.
- Normalization: Pixel intensities were normalized to a [0, 1] range by dividing by 255 to stabilize and accelerate the model training process.

**Figure 5:**
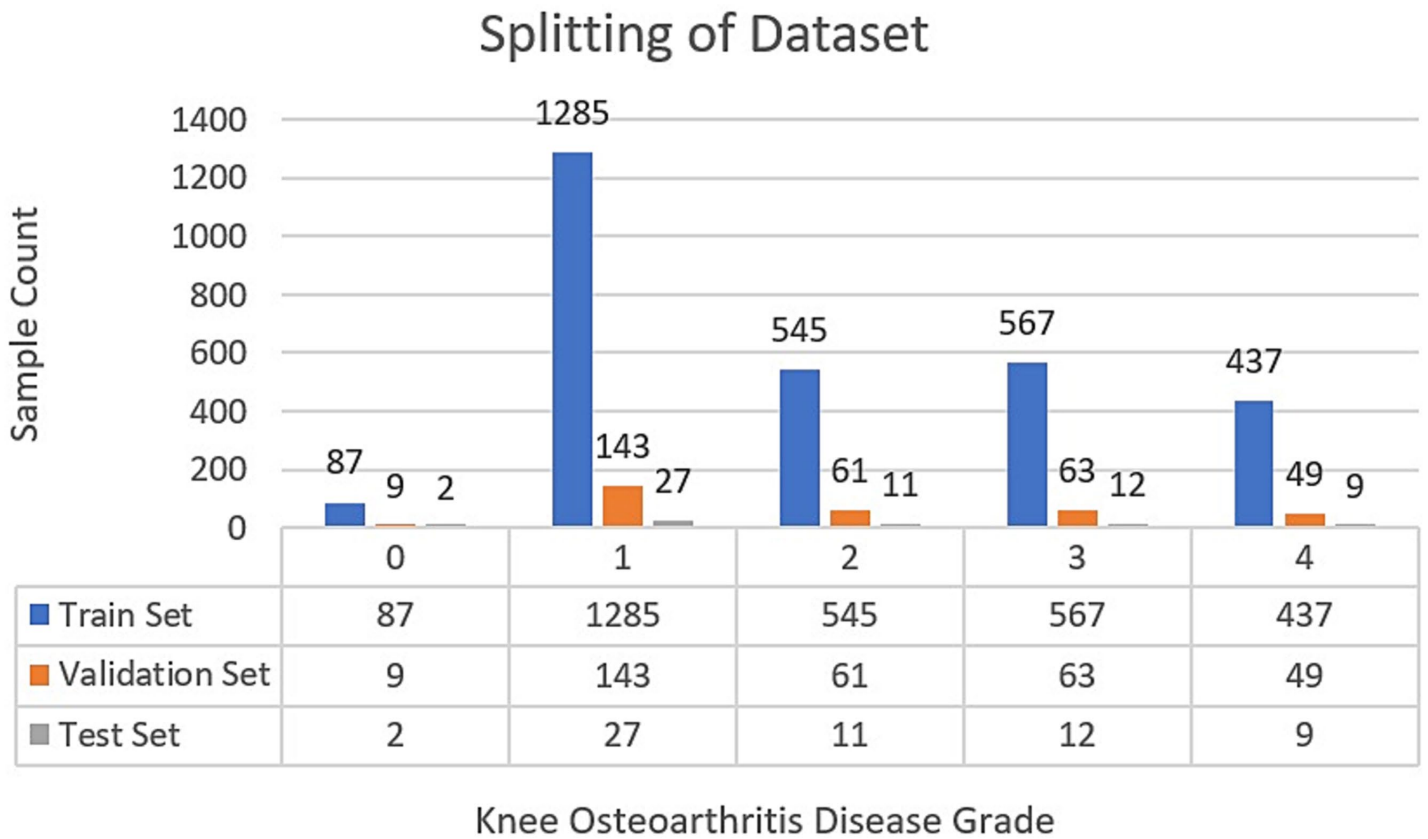
Splitting of the dataset after augmentation.

The code for preprocessing is given in^1^. The final, processed dataset of individual knee images was then split for model development, as detailed in Figure 5.

### 2.7 Splitting of data and augmentation

**Figure 6:**
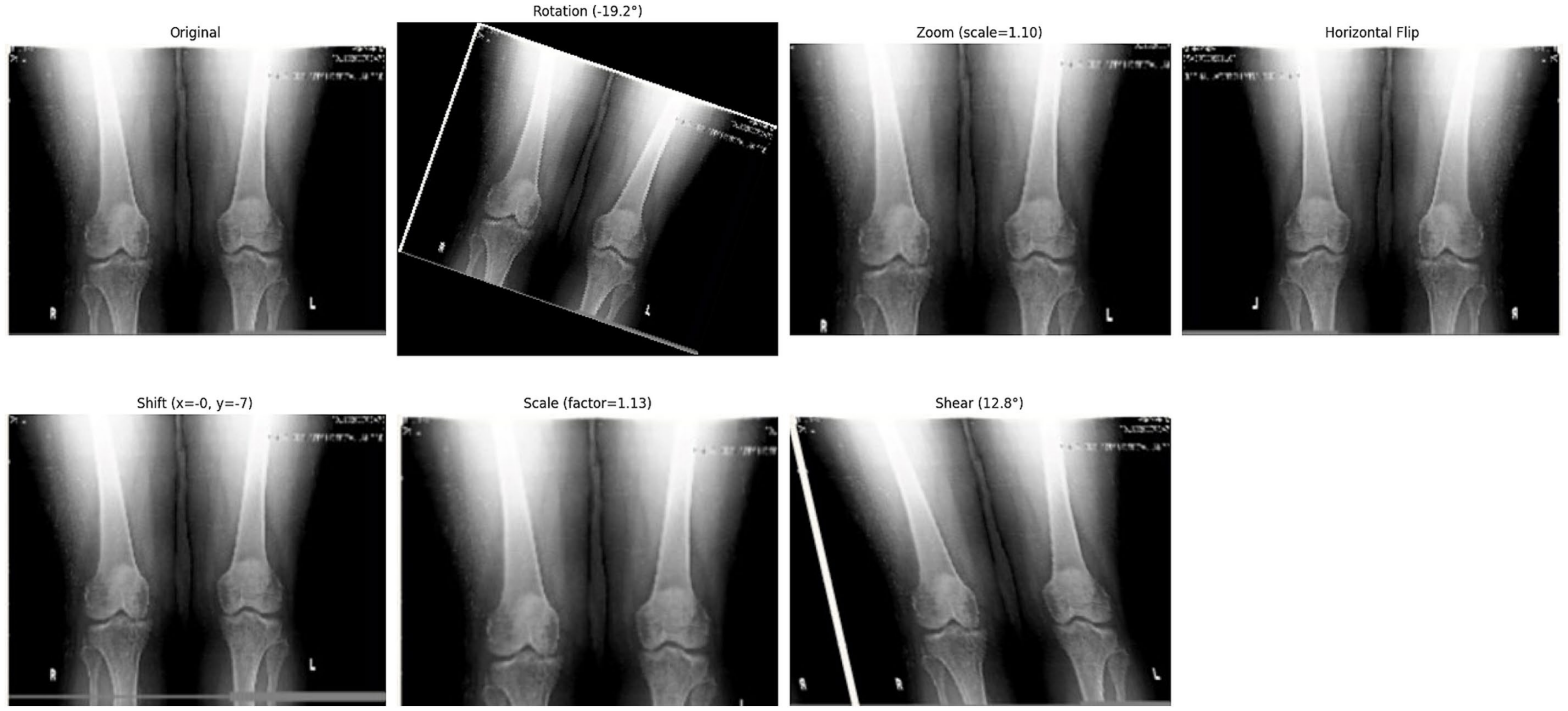
Effect of different augmentation techniques on a sample image.

Following the completion of the pre-processing procedures, the dataset was divided into three separate sets: train, test, and validation. This partitioning was achieved via a two-step approach. Initially, 80% of the data was allocated for model training, with the remaining 20% (61 samples) designated for testing. In the subsequent phase, the training dataset underwent augmentation through rotation, zoom, and affine transformation techniques. Images were randomly rotated within a range of ±30 degrees to introduce rotational invariance. Zoom augmentation was applied by scaling images with a random factor between 0.8 and 1.2, enabling the model to handle variations in object size and perspective better. Additionally, affine transformations were employed, incorporating horizontal flipping, random shifts up to 10% of the image dimensions, scaling within the same 0.8–1.2 range, and shear angles up to ±20 degrees. Figure 6 shows the resulting images after applying different augmentation techniques to a sample image. The complete code for data augmentation is also given in ^1^. These parameters were chosen to increase dataset variability without distorting critical anatomical features relevant to KL grading. This augmented training dataset was further divided into 90% training and 10% validation. The number of training samples after data augmentation and testing sample (with no augmentation) per class is depicted in Figure 5.

### 2.8 Network architecture

**Figure 7:**
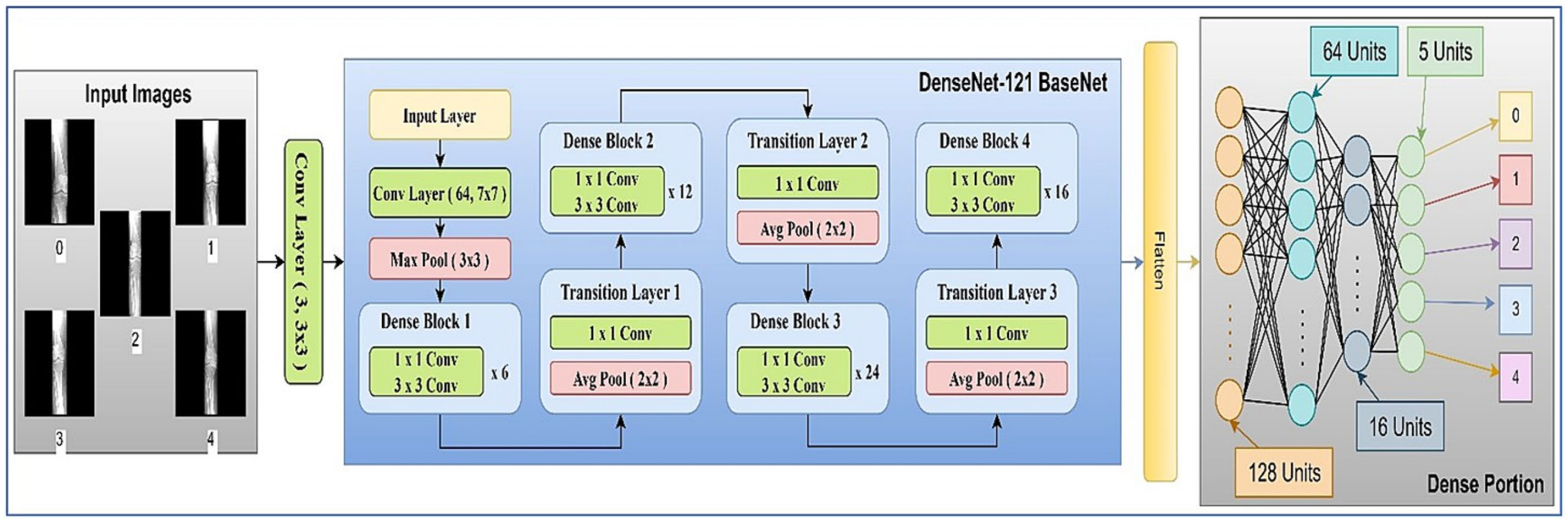
Architecture diagram of proposed work.

The main components used in the architecture are discussed in the following subsections. The model architecture consisted of pre-trained 2D convolutional layers followed by additional layers. Figure 7 represents the visual representation of the model.

#### 2.8.1 Architecture block

The network architecture of the proposed model is shown in Figure 7. It contained an initial layer of 2D convolution with an activation of the Rectified Linear Unit (ReLU) function, which converts the images to a shape of 224, 224, 3: from 224, 224, 1 so that the images can be fed into the transfer learning (TL) model of DenseNet121 (29). DenseNet121 is a pre-trained model on a large dataset containing weights that are useful in the training process.

In TL, the pre-trained model is typically referred to as the base model. The model was implemented using TensorFlow and the Keras library. The base DenseNet-121 architecture, pre-trained on ImageNet, was used without freezing any layers, allowing for full fine-tuning on our medical image dataset (30). This approach enables the model to adapt its low-level feature extractors (e.g., edge and texture detectors) to the specific characteristics of X-ray images. The custom classification head was trained from scratch. We employed the Adam optimizer for its adaptive learning rate capabilities and used a reduced learning rate to facilitate stable fine-tuning. A learning rate scheduler and early stopping were implemented to prevent overfitting and optimize training time. To mitigate overfitting, we applied L2 weight regularization (*λ* = 1 × e^−4^) to the kernel weights of all fully connected layers, combined with Dropout (rate = 0.3) after each hidden layer activation. The architecture diagram of the proposed model is shown in Figure 7. The summary of the model layers and trainable/non-trainable parameters is shown in Table 1.

**Table 1:** Summary of the proposed model.

Layer	Input shape	Output shape	Parameters
Input layer	(None, 224, 224, 1)	(None, 224, 224, 1)	0
Convolutional layer	(None, 224, 224, 1)	(None, 224, 224, 3)	30
**DenseNet121 (BaseNet)**	**(None, 224, 224, 3)**	**(None, 7, 7, 1,024)**	**7,037,504**
Flatten	(None, 7, 7, 1,024)	(None, 50,176)	0
FC_1	(None, 50,176)	(None, 128)	6,422,656
Activation (ReLU)	(None, 128)	(None, 128)	0
FC_2	(None, 128)	(None, 64)	8,256
Activation (ReLU)	(None, 64)	(None, 64)	0
FC_3	(None, 64)	(None, 16)	1,040
Activation (ReLU)	(None, 16)	(None, 16)	0
FC_4	(None, 16)	(None, 5)	85
Activation (SoftMax)	(None, 128)	(None, 128)	0
Total parameters	13,469,571		
Trainable parameters	13,385,923		
Non-trainable parameters	83,648		

### 2.9 2D-convolutional layer

The 2D convolutional layer is a crucial component of convolutional neural networks (CNNs) that excels in extracting spatial characteristics from input images (31). In the forward pass, the convolution procedure consists of sliding a small filter over the input image, computing element-wise products between filter weights and corresponding image pixels, and summing these products to create a feature map as defined in Equation 1. This method catches local patterns and identifies pertinent characteristics. The backward pass, also known as backpropagation, calculates gradients with respect to the loss function as represented by Equations 2, 3; enabling the network to adjust its training parameters accordingly.


(1) $$y_{i,j}=\sum_{m=0}^{M-1}\sum_{n=0}^{N-1}X_{i+m,\;j+n}.W_{m.n}+b$$

Where, $y_{i,j}$ is the output feature map value at the position (i,j) . M and N represent the filter dimensions, W is the weight at position (m,n) and b is the bias term.


(2) $$\frac{\mathrm{\delta L}}{\delta X_{i,j}}=\sum_{m=0}^{M-1}\sum_{n=0}^{N-1}\frac{\mathrm{\delta L}}{\delta Y_{i-m,j-n}}.W_{m.n}\;$$

(3) $$\frac{\mathrm{\delta L}}{\delta W_{m,n}}=\sum_{i=0}^{H-1}\sum_{j=0}^{W-1}\frac{\mathrm{\delta L}}{\delta Y_{i,j}}.X_{i+m.j+n}\;$$

Where, H and W are the height and width of the output feature map. $\delta X_{i,j}$ represents the gradient loss relative to the output feature map.

### 2.10 Fully connected layer

In a neural network, a fully connected (FC) layer connects every node from the previous layer to each node in the current layer (32). This layer performs a linear transformation on the input, followed by the application of an activation function. The equation for the FC (33) layer is represented as shown in Equation 4.


(4) y=f(∑Wx+b)

Where x is the input vector, W is the weight matrix, b is the bias vector, f is the activation function, and y is the output vector.

### 2.11 Evaluation metrics

The performance of the proposed model was evaluated using various metrics. To benchmark the model’s effectiveness, we employed four commonly used evaluation metrics: accuracy, area under the curve (AUC), recall, and precision. These metrics are widely used to assess the performance of classification models.

#### 2.11.1 Accuracy

In this study, five classes were used for classification, and categorical accuracy was employed to evaluate the model’s accuracy. Equation 5 represents a mathematical representation of the accuracy (32) metric.


(5) $$\text{Accuracy}=\frac{\mathrm{TP}+\mathrm{TN}}{\mathrm{TP}+\mathrm{TN}+\mathrm{FP}+\mathrm{FN}}$$

Where TP , FP , TN , and FN stand for True Positive, False Positive, True Negative, and False Negative, respectively.

#### 2.11.2 Area under curve

The Area Under the Curve (AUC) evaluation metric was also used to measure the model’s performance. The mathematical representation of AUC is depicted in Equation 6 (34, 35).


(6) $$\mathrm{AUC}=\int_{0}^{1}\mathrm{TPR}(\mathrm{FP}R^{-1}(t))\mathrm{dt}$$

Where, TPR is the true positive rate, FPR is the false positive rate, $\mathrm{FP}R^{-1}$ and is the inverse function of the FPR at threshold t . The AUC value ranges between 0 and 1, with higher values indicating better performance.

#### 2.11.3 Recall

In this study, recall was used in conjunction with other evaluation metrics. Also referred to as sensitivity or the true positive rate, recall measures the model’s ability to identify positive instances accurately. It is calculated as the ratio of true positive predictions to the total number of actual positive samples. The formula for recall (33, 36) is shown in Equation 7.


(7) $$\text{Recall}=\frac{\mathrm{TP}}{\mathrm{TP}+\mathrm{FN}}$$

#### 2.11.4 Precision

Precision (33, 37, 38), also known as specificity, was calculated to assess the proportion of true positive predictions made by the model relative to all positive predictions. The mathematical formula for precision is provided in Equation 8.


(8) $$\text{Precision}=\frac{\mathrm{TP}}{\mathrm{TP}+\mathrm{FP}}$$

#### 2.11.5 Categorical cross-entropy loss

Categorical Cross-Entropy (CCE) (39) loss was used to measure the difference between the model’s predicted output and the actual target values. The formula for categorical cross-entropy loss is provided in Equation 9.


(9) $$L(y,\hat{y})=-\sum_{i=1}^{C}y_{i}\times \log\hat{y_{i}}$$

Where, y is the true label, $\hat{y}$ is the predicted probability (ranging from 0 to 1), and C is the number of classes in the dataset.

## 3 Experimentation and results

The tests in this study were carried out on a server with specific hardware: 64GB of RAM, a 5GB NVIDIA QUADRO P2000 Graphics Processing Unit (GPU), and a 256GB Hard Disk. To evaluate the model’s performance, the test set was created by splitting the dataset before augmentation and training. Using various metrics ensures that the model is robust and reliable from multiple perspectives. When we combine all of these outcomes, we can see how successfully the model was trained. Other variables, such as loss and overfitting, also play a role in this process.

Several experiments were conducted on the dataset, involving two distinct preprocessing approaches. Initially, the model was trained using images of separated knees, while the second approach involved adding annotation boxes to these images. In both cases, transfer learning techniques were employed, incorporating hyperparameter tuning and regularization strategies to enhance performance. Various scenarios were explored to address concerns of overfitting and underfitting. Grade-0 and grade-1 X-ray views were identical; however, they were assigned different labels based on patient-reported pain in the knee. In cases where patients experience pain and the X-ray appears normal, they are categorized as grade-1. Conversely, when there was no pain and the X-ray was normal, it was classified as grade-0, serving as the ground truth label. This intricacy contributed to the challenge of training the dataset. Due to the unsatisfactory results of the object detection trials, testing on this dataset was discontinued. Further details of these experiments are elaborated upon in the subsequent section.

### 3.1 Proposed model

Table 2 presents the results of the tests conducted. Each test experiment contained a different architecture or a change in the optimizer. The “Model” column displays information about the transfer learning models used as BaseNet, and the “FC layers” column indicates the number of fully connected layers employed after the BaseNet. The further columns represent the testing values of loss, accuracy, AUC, precision, and recall scores for the given experiment.

**Table 2:** Proposed 3D model test results.

Sr. No	Model	FC layers	Parameters	Loss	Acc%	AUC%	Precision%	Recall%
1	ResNet50-V2	3	36.41 m	2.8311	54.10	79.91	55.93	54.10
2	ResNet50	3	36.44 m	3.7643	60.66	83.03	60	59.02
3	Inception-ResNetV2	3	56.807 m	3.3459	55.37	80.70	58.77	55.37
4	DenseNet201	4	24.355 m	2.2322	60.66	80.92	60.66	60.66
5	MobileNet	3	9.66 m	2.1614	60.66	83.65	61.02	59.02
6	DenseNet121	3	10.26 m	2.2285	57.38	81.44	56.67	55.74
7	DenseNet121	3	13.46 m	2.382	68.85	85.67	68.33	67.21

The proposed model was compared with other state-of-the-art models. Other networks performed well on most of the datasets used, utilizing their trained weights. There are several famous network architectures, including DenseNet, ResNet-V2, MobileNet, MobileNet-V2, VGG16, VGG19, EfficientNet, and many others. There are other machine learning approaches, such as SVM, random forest, decision tree, and several others. We mentioned most of our experimental results, which achieved accuracies near the highest ones.

### 3.2 Machine learning approaches

Machine Learning (ML) is the traditional approach. Algorithms in ML, such as SVM, Random Forest, and Decision Trees, are powerful tools that enable computers to learn patterns and make predictions from data. SVM aims to find the best boundary between different classes, maximizing the margin between them. Random Forest combines multiple decision trees to make more accurate predictions by reducing overfitting. Decision Trees work by making a series of if-else decisions based on features to arrive at an outcome. These algorithms excel in various tasks like classification, regression, and anomaly detection. By learning from historical data, they can generalize and make predictions on new, unseen data, making them invaluable for a wide range of real-world applications.

The preprocessed images transformed 1D vectors, enabling them to be compatible with various machine-learning algorithms. Further, these 1D vectors were normalized using standard scaling to enhance algorithm compatibility. The performance evaluation of these algorithms is outlined in Table 3.

**Table 3:** ML algorithms results and description of parameters.

Sr. No	Algorithm	Accuracy%	Details
1	Decision tree	50.81	Criterion = entropy
2	SVM	54.09	L2 penalty = 0.5, decision function = ovo
3	Random forest	55.73	Criterion = entropy, n-estimator = 500

### 3.3 Evaluation metric comparison

**Figure 8:**
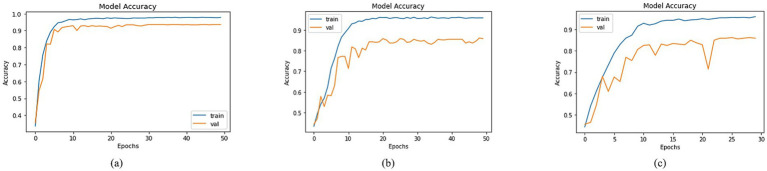
Accuracies of trained models with **(a)** DenseNet201, **(b)** MobileNet and **(c)** DenseNet121 as BaseNet.

By employing the accuracy formulas specified in the accuracy section, a comparison of accuracy was conducted across different models. Most of the approaches achieved training accuracies surpassing 90% and validation accuracies over 80%. To prevent data leakage issues, the validation set was derived from the augmented dataset, while the test set was separated from the original dataset. Among the models, the top three testing accuracies were observed with DenseNet201, MobileNet, and DenseNet121 as the Basenet. In Figure 8, graphs illustrating training and validation curves displayed accuracy values across epochs. In Figure 8, DenseNet201 exhibited a training accuracy of 97.79% and a validation accuracy of 93.59%. In comparison, MobileNet and DenseNet-121 achieved training accuracies of 95.89 and 95.93%, respectively, as shown in Figures 8b,c, accompanied by validation accuracies of 85.85% for both.

**Figure 9:**
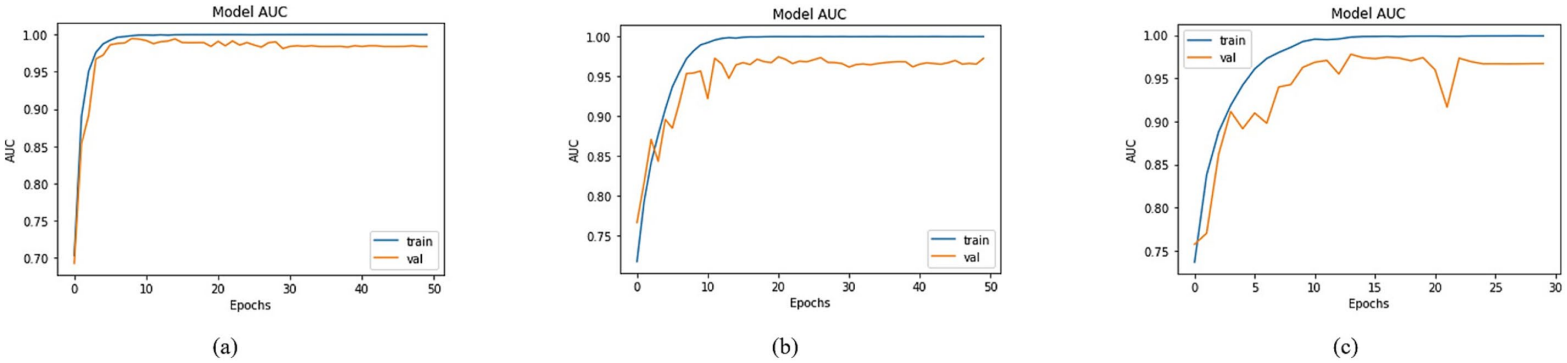
AUC of trained models with **(a)** DenseNet201, **(b)** MobileNet and **(c)** DenseNet121 as BaseNet.

Utilizing the equations outlined in the AUC section, a comprehensive analysis was conducted to compare the AUC across various models using distinct BaseNet configurations. The visual depiction in Figure 9 of the training and validation curves over epochs provides a comprehensive insight into the models’ progress, highlighting their respective AUC values. The training Area Under the Curve (AUC) figures were notably high, registering at 99.97, 99.92, and 99.90% for DenseNet201, MobileNet, and DenseNet-121, in Figures 9a–c, respectively. Correspondingly, validation AUC values reached 98.30, 97.19, and 96.69% for the respective models. This affirmation of high AUC values signifies the models’ strong predictive capabilities and robust learning representations, suggesting their potential for accurate classification tasks.

**Figure 10:**
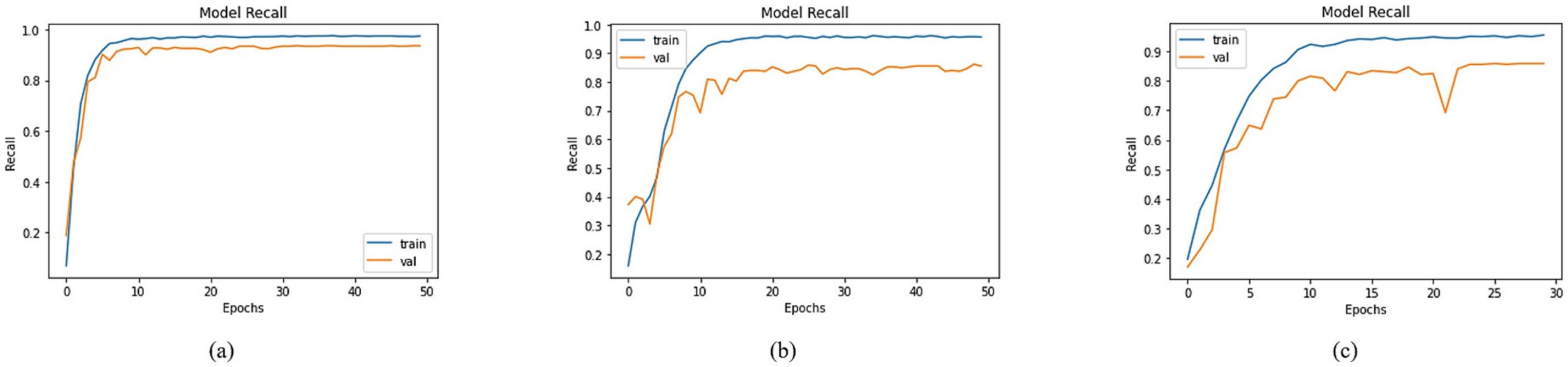
Recall of trained models with **(a)** DenseNet201, **(b)** MobileNet and **(c)** DenseNet121 as BaseNet.

An extensive evaluation of recall was undertaken, involving diverse models. The visualization of training and validation curves in Figure 10, across successive epochs, effectively portrays the models’ learning trajectories and their corresponding recall values. Among the models examined, including DenseNet-201, MobileNet, and DenseNet-121, distinct recall trends emerged. In Figure 10, DenseNet201 exhibited a training recall of 97.28% and a validation recall of 93.42%. In contrast, MobileNet demonstrated training and validation recalls of 95.65 and 85.54%, respectively, as shown in Figure 10. For DenseNet-121, the training recall stood at 95.48%, while the validation recall was registered at 85.85%, as shown in Figure 10. These recall values provided valuable insights into the models’ capacity to correctly identify positive instances, thereby contributing to a nuanced understanding of their performance dynamics within classification tasks.

**Figure 11:**
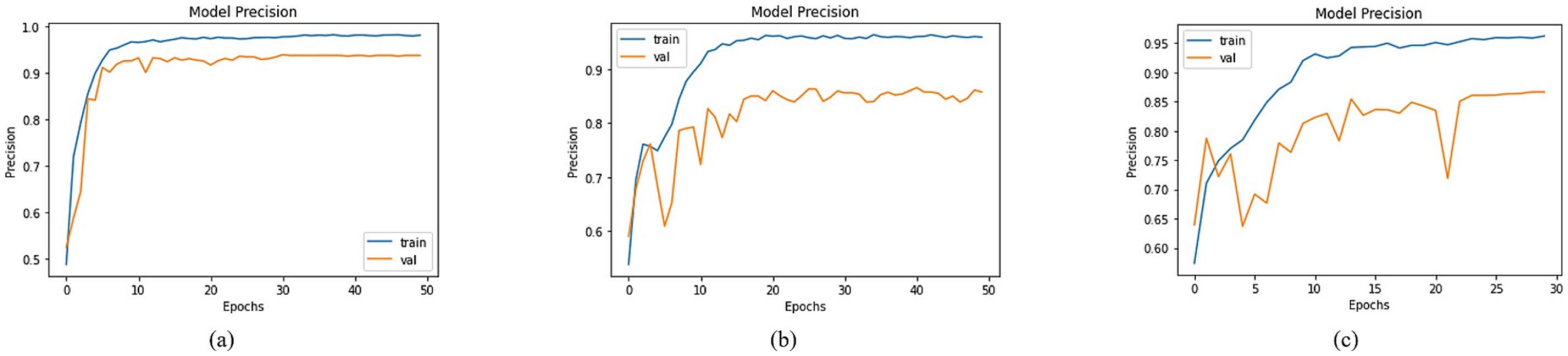
Precision of trained models with **(a)** DenseNet201, **(b)** MobileNet and **(c)** DenseNet121 as BaseNet.

An in-depth assessment of precision was conducted, encompassing diverse models with varied base network configurations. Visualizing training and validation curves across multiple epochs effectively illustrated the models’ learning trajectories alongside their corresponding precision values in Figure 11. In this context, DenseNet201, MobileNet, and DenseNet121 exhibited distinct precision trends. Specifically, DenseNet201 achieved a training precision of 97.98% and a corresponding validation precision of 93.74%, as shown in Figure 11. For MobileNet, the training and validation precisions were 95.98 and 85.80%, respectively, as depicted in Figure 11. In the case of DenseNet121, a training precision of 96.21% was recorded, accompanied by a validation precision of 86.65% as shown in Figure 11. These precision values provided key insights into the models’ ability to make accurate positive predictions, thereby shedding light on their performance dynamics across classification tasks.

**Figure 12:**
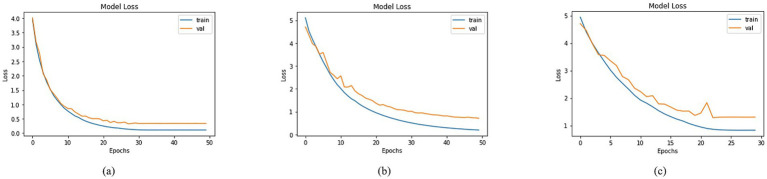
Loss of trained models with **(a)** DenseNet201, **(b)** MobileNet and **(c)** DenseNet121 as BaseNet.

A comprehensive analysis was conducted to compare the loss across different models utilizing distinct basenet configurations. Graphs depicting training and validation curves over successive epochs effectively conveyed the models’ learning progress in Figure 12, accompanied by their corresponding loss values. Among the models, DenseNet201, MobileNet, and DenseNet121 demonstrated varying loss figures. Specifically, DenseNet201 demonstrated a training loss of 0.1090 and a validation loss of 0.3383, whereas MobileNet showed training and validation losses of 0.1953 and 0.7102, as illustrated in Figure 12. DenseNet121 recorded a training loss of 0.8255 and a validation loss of 1.3024, as shown in Figure 12. These diverse loss values reflected the models’ convergence and performance characteristics, ultimately contributing to the understanding of their efficacy in addressing classification tasks.

### 3.4 Evaluation

The training and validation evaluations of the proposed model have been detailed in the preceding sections. Below are the testing accuracies of several additional models, providing a comprehensive breakdown of our experimental results. Essential details concerning the test set are provided in Table 2. Under the “Model” column, BaseNet names are listed, while the “FC Layer” count enumerates the number of fully connected layers utilized to post the BaseNet. The “Parameters” column indicates the total parameters (trainable & non-trainable) within each model. Notably, in the case of DenseNet121 at the 6th entry, although 3FC layers were employed, the units within these layers underwent modification. Significantly, the 7th entry corresponds to the proposed model, as featured in Figure 7. This table serves as a comprehensive reference, outlining the key characteristics and configurations of the experimented models.

## 4 Discussion

Our study demonstrates that a transfer learning approach based on DenseNet-121 can effectively grade knee osteoarthritis from X-ray images with a clinically useful level of accuracy (68.85%). A key differentiator of our method was the automated isolation of individual knees from bilateral X-rays, a crucial preprocessing step that moves beyond using pre-segmented public datasets. Despite challenges such as class imbalance and the subtle radiographic differences between KL grades 0 and 1, our model outperformed both other deep learning architectures and traditional machine learning algorithms.

Our proposed DenseNet-121 model achieved the best performance (68.85% accuracy), likely due to its efficient feature reuse and gradient flow, which is beneficial for training on smaller datasets. In contrast, larger models like DenseNet201 may have overfitted, while shallower models like MobileNet lacked the representational capacity. The key novelty of our work lies not only in this comparative analysis but also in developing an end-to-end pipeline that starts with raw bilateral X-rays, addressing the clinically relevant task of automatic knee isolation—a step often bypassed in studies using pre-curated, single-knee datasets.

Dataset size and balance also strongly influence results. While large, multi-thousand-sample repositories enable deeper models to generalize effectively, our dataset of 602 knees was relatively small and imbalanced, particularly between grades 0 and 1. This overlap in radiographic appearance makes classification inherently difficult and contributes to reduced performance for some architectures. Preprocessing strategies further affected outcomes. Previous studies often employed curated datasets with manually segmented knees, which simplifies the classification task. By contrast, our automated knee isolation pipeline introduced real-world variability, including zero-padding and normalization, which makes the approach more clinically relevant but also slightly more error-prone compared to pre-segmented datasets. Although confidence intervals, statistical significance testing, and k-fold cross-validation were not performed due to dataset constraints, these analyses are planned for future work as the dataset is expanded.

Finally, differences in imaging modalities should be considered. MRI-based studies generally outperform X-ray–based studies in detecting early KOA, as MRI provides detailed cartilage-level information that X-rays cannot capture. This explains why AUC values reported in MRI studies often exceed 90%, whereas our X-ray–based approach achieved a strong but lower AUC of 85.67%. Collectively, these factors underscore that model performance is shaped not only by the choice of architecture but also by the nature of the dataset, preprocessing pipeline, and imaging modality.

Together, these findings highlight that model performance in KOA diagnosis is not solely determined by architecture but is significantly shaped by dataset characteristics, preprocessing approaches, and modality-specific features.

The novelty of this study lies in several aspects that distinguish it from existing work on KOA diagnosis. First, we developed an automated image-processing pipeline for knee isolation from bilateral radiographs, a step often overlooked in prior studies that relied on pre-segmented or manually curated datasets. By automating this process, our approach brings AI-based diagnosis closer to real-world clinical applicability, where radiographs are rarely preprocessed. Second, we curated a new dataset comprising 602 knee images from 301 patients at the Social Security Teaching Hospital, Lahore, representing one of the first such collections from Pakistan. This dataset adds valuable diversity to the global research landscape and provides a benchmark for populations that have been underrepresented in KOA studies. Third, we carried out a systematic comparative evaluation of both classical machine learning algorithms and several deep learning architectures, identifying DenseNet-121 as the most effective in this challenging setting. Importantly, our dataset included a clinically challenging cohort where KL-0 cases were not healthy, asymptomatic controls, but rather symptomatic patients with normal X-rays. This design makes the classification task more complex but also more clinically realistic, as it mirrors the diagnostic challenges faced by practitioners. Together, these contributions highlight the originality of our work and its potential to advance AI-based diagnostic tools for KOA.

An important consideration in our study is the potential for spectrum bias. Our inclusion criteria required participants to have persistent knee pain. Consequently, our ‘KL-0’ group represents a challenging cohort of symptomatic controls—patients with pain but no radiographic abnormalities—rather than truly healthy, asymptomatic individuals. This likely contributed to the model’s difficulty in distinguishing between KL-0 and KL-1 grades, as the defining clinical symptom (pain) was present in both groups, leaving only subtle radiographic clues for the model to learn.

Two factors primarily constrained the model’s performance. First, the fundamental clinical challenge of distinguishing between KL grade 0 (no pain, normal X-ray) and grade 1 (presence of pain, normal X-ray) based solely on radiographic features led to predictable misclassifications. Second, the necessary use of zero-padding to standardize image sizes after knee isolation may have introduced a background bias, a trade-off against the benefits of our automated segmentation pipeline.

Several prior studies have explored KOA classification with both machine learning and deep learning approaches, reporting varying performance depending on dataset size, preprocessing, and imaging modality. Abdullah et al. (40) proposed a deep learning approach using ResNet50 trained on a much larger dataset of 3,172 knee X-rays. Their model achieved higher accuracy than ours; however, their dataset was pre-curated and balanced, whereas our dataset of 602 knees was collected directly from a clinical setting and included symptomatic KL-0 cases. This distinction makes our classification task inherently more challenging but also more reflective of real-world diagnostic conditions. Ahmed et al. (41) explored segmentation techniques with both traditional ML and DL, highlighting the benefits of multimodal data integration for improving performance. While their work demonstrates the value of data enrichment, our pipeline focused on automatic segmentation of raw bilateral radiographs, offering a pathway toward clinical applicability where such pre-curated data are not readily available.

Ashinsky et al. (42) reported 75% accuracy using a WND-CHRM model on a small MRI dataset of only 68 subjects. Their use of cartilage maps provided rich soft-tissue detail, which is not available in radiographs, explaining their higher performance despite the small sample size. Yeoh et al. (43), in a review of 74 studies, emphasized that many reported high accuracies rely on curated datasets or 3D CNNs, but also noted the challenges in translating such methods into routine clinical practice. Similarly, Olsson et al. (44) achieved an AUC of 80% using the ResNet model. However, their study did not systematically compare multiple architectures as we did, and their dataset was larger and less clinically ambiguous than ours. Thomas et al. (45), who utilized DenseNet for KOA classification, reported an accuracy of 60%, which is lower than our best accuracy of 68.85%. However, their study lacked a comparative evaluation and omitted computational considerations.

Our selection of DenseNet-121 was deliberate for several key reasons relevant to KOA classification: (1) Its dense connectivity pattern promotes feature reuse, which is crucial when training data is limited, as in our medical imaging context; (2) The model’s efficient parameter use reduces overfitting risk compared to similarly deep architectures; (3) The strong performance on ImageNet suggests robust feature extraction capabilities that transfer well to radiographic texture analysis. While we acknowledge that ablation studies would provide more definitive evidence, our comprehensive comparison against multiple architectures (Table 2) demonstrates DenseNet-121’s superior empirical performance for this specific task. From a deployment perspective, despite its depth, the DenseNet-121 model maintained inference time averaged at 0.15 s per knee image, making real-time batch processing feasible in clinical workflows on our test hardware (NVIDIA QUADRO P2000).

Although our model achieved an accuracy of 68.85%, it represents a strong proof of concept for an automated grading pipeline. This level of performance is not sufficient for the system to be adopted as an independent diagnostic tool in clinical practice. Instead, the present study should be viewed as developing an auxiliary computer-aided diagnostic system, designed to complement rather than replace clinical expertise. Note that Early diagnosis of KOA is critical because treatment options become increasingly limited and invasive as the disease progresses. In advanced stages, surgical interventions such as high tibial osteotomy combined with chronic distraction tissue regeneration and computer-assisted external fixation have been explored to correct severe joint deformities (46). In this capacity, the model can serve as a decision-support tool, offering consistent preliminary grading that may help junior radiologists and medical trainees reduce interpretation variability and improve confidence in early diagnosis. With further development—including the expansion of dataset size, mitigation of class imbalance, and integration of multimodal data such as MRI and clinical history—the system’s performance may approach the reliability required for standalone diagnostic use.

## 5 Conclusion

This study presented an end-to-end, AI-based CAD system for grading Knee Osteoarthritis from raw bilateral X-ray images. Our key contribution is the development of a fully automated pipeline that segments individual knees—a crucial step for precise analysis—before classification. Through a comprehensive comparative analysis, we demonstrated that a transfer learning approach using DenseNet-121 is the most effective, achieving a test accuracy of 68.85% and an AUC of 85.67% on a challenging, five-class, imbalanced dataset. While this performance is a promising proof of concept, it highlights the complexity of fine-grained KOA grading and the difficulty in distinguishing early disease stages. This system shows potential as a valuable assistive tool for training and standardizing assessments, paving the way for more accessible and consistent KOA diagnosis.

## 6 Limitation

Although our approach showed some potential, this study has several limitations. Firstly, despite its diversity, the dataset utilized may still lack coverage of particular demographic groups. Additionally, the dataset was imbalanced, which could impact the model’s performance and reliability. Secondly, the model’s performance may vary with different X-ray imaging equipment and settings, which should be considered for real-world applications. Lastly, the model’s ability to generalize to other medical image analysis tasks beyond KOA classification remained unexplored and should be a subject of future investigation.

Additionally, k-fold cross-validation was not applied due to dataset constraints, as repeated partitioning risked class depletion in minority grades. Future work will incorporate stratified k-fold validation and external dataset testing to assess generalizability across institutions.

## 7 Future work

The imbalanced dataset, with a disproportionately low number of KL-0 cases compared to KL-1, is a main concern in this study. This imbalance inevitably impacts robustness and generalizability, particularly in distinguishing early KOA stages. While our results demonstrate the feasibility of automated KOA grading in a real-world clinical cohort, future work should address class imbalance through strategies such as oversampling, class-weighted loss functions, or synthetic data augmentation. Future work could explore advanced deep learning architectures and ensemble techniques to bolster results further and address nuanced challenges in KOA detection. The dataset’s diversity is a strength; however, expanding it to include more demographics and imaging scenarios would further enhance generalization.

For future work, explainable AI (XAI) techniques, such as Grad-CAM and layer-wise relevance propagation, can be incorporated to enhance model interpretability. These visual explanations will help identify the specific radiographic regions that influence model predictions, providing clinicians and radiologists with greater confidence in the system’s decision-making process. Incorporating such interpretability tools will also support clinical validation and facilitate integration of AI-assisted diagnosis into real-world medical workflows.

## Data Availability

The raw data supporting the conclusions of this article will be made available by the authors, without undue reservation.

## Footnotes

^1^ https://drive.google.com/drive/folders/1Hbz5q0P7GS6e4sOB7LklbJvvD75qLTHl
